# Sex Differences Linking Pain-Related Fear and Interoceptive Hypervigilance: Attentional Biases to Conditioned Threat and Safety Signals in a Visceral Pain Model

**DOI:** 10.3389/fpsyt.2020.00197

**Published:** 2020-03-24

**Authors:** Franziska Labrenz, Sopiko Knuf-Rtveliashvili, Sigrid Elsenbruch

**Affiliations:** Institute of Medical Psychology & Behavioral Immunobiology, University Hospital Essen, University of Duisburg-Essen, Essen, Germany

**Keywords:** gut-brain axis, visceral pain, attentional bias, hypervigilance, pain-related fear, anxiety, sex differences

## Abstract

Although the broad role of fear and hypervigilance in conditions of the gut-brain axis like irritable bowel syndrome is supported by converging evidence, the underlying mechanisms remain incompletely understood. Even in healthy individuals, it remains unclear how pain-related fear may contribute to pain-related attentional biases for acute visceral pain. Building on our classical fear conditioning work in a clinically relevant model of visceral pain, we herein elucidated pain-related attentional biases shaped by associative learning in healthy women and men, aiming to elucidate possible sex differences and the role of psychological traits. To this end, we compared the impact of differentially conditioned pain-predictive cues on attentional biases in healthy women and men. Sixty-four volunteers accomplished a visual dot-probe task and subsequently underwent pain-related fear conditioning where one visual cue (CS^+^) was contingently paired with a painful rectal distention (US) while another cue remained unpaired (CS^−^). During the following test phase, the dot-probe task was repeated to investigate changes in attentional biases in response to differentially valenced cues. While pain-related learning was comparable between groups, men revealed more pronounced attentional engagement with the CS^+^ and CS^−^ whereas women demonstrated stronger difficulties to disengage from the CS^+^ when presented with a neutral cue. However, when both CS^+^ and CS^−^ were presented together, women revealed stronger difficulties to disengage from the CS^−^. Regression analyses revealed an interaction of sex, with negative affect predicting stronger avoidance of the CS^+^ and stronger difficulties to disengage attention from the CS^−^ in men. These results provide first evidence that pain-related fear conditioning may induce attentional biases differentially in healthy women and men. Hence, sex differences may play a role in attentional mechanisms underlying hypervigilance, and may be modulated by psychological vulnerability factors relevant to chronic visceral pain.

## Introduction

Fear is a potent motivator that drives learning and behavior. It serves to rapidly shift attention toward signals of threat in order to avoid bodily harm, engage in self-protection and seek safety. Although adaptive by nature, fear and ensuing avoidance behaviors can also become maladaptive, with broad implications for the pathophysiology and treatment of chronic pain (1, 2). The fear-avoidance model of pain proposes a vicious cycle of pain-related fear, hypervigilance and avoidance, maintained, and modulated by psychological vulnerability factors like anxiety, catastrophizing, and negative affect (3, 4). Pain-related fear is essentially governed by the principles of classical and instrumental conditioning, engaging attentional resources that are relevant to hypervigilance, and avoidance. As an inherently fear-evoking stimulus, pain attracts strong attentional responses leading to increased sensitivity for negative information and aberrant attentional orienting toward threat (5, 6). These responses involve opposing, yet inextricably linked mechanisms of facilitated engagement toward threat, avoidance and difficulties to disengage with individuals orienting toward or away from threat early during attentional processing and subsequent avoidance at later stages (7, 8). While these processes are broadly established in the context of anxiety and posttraumatic stress (9, 10), evidence in the context of pain-related fear remains scarce and inconsistent (6, 11), especially with regard to interoceptive, visceral pain. Visceral pain is of high clinical relevance, specifically in conditions of the gut-brain axis like irritable bowel syndrome (IBS). Compared to other types of exteroceptive pain, visceral pain is considered to be characterized by a unique biological salience, as supported by greater visceral pain-related fear and enhanced pain-related learning (12, 13), making this a suitable preclinical model to study pain-related attentional biases. While numerous studies addressed attentional biases toward threat only, safety cues signaling the absence of an aversive event were also found to induce attentional biases (14) and are prioritized under threatening conditions (15). Specifically in visceral pain, patients (16, 17) and healthy individuals (18) demonstrated enhanced awareness of safety cues suggesting that attentional biases may also pertain to safety cues.

Building on our conditioning work in a clinically relevant model of visceral pain (19–21), we herein aimed to elucidate attentional biases induced by conditioned pain-related fear and safety signals in healthy volunteers. Inspired by broad knowledge about higher prevalence and incidence of chronic visceral pain in women (22), initial evidence suggesting sex differences in pain-related fear conditioning (23) and attentional coping strategies (24), we a priori committed to elucidating sex differences. Moreover, pain-related attentional biases seem to vary as a function of inter-individual differences in anxiety sensitivity and catastrophizing, but findings are heterogeneous (22, 25–27).

We herein employed a visual dot-probe task to address whether conditioning results in attentional biases related to attentional avoidance, facilitated engagement and/ or difficulties to disengage from pain-related threat and safety cues. We hypothesized that women would demonstrate more pronounced attentional biases toward threat cues, reflected by stronger engagement at short stimulus durations, as well as stronger difficulties to disengage at longer stimulus durations. Moreover, we assumed that women would demonstrate more pronounced biases to safety cues as conditioned inhibitors of fear responses and a clinically relevant phenotype in anxiety-related disorders (28). Finally, we explored the sex-specific role of psychological vulnerability factors as predictors, specifically aiming to assess a greater contribution of anxiety, negative affect as well as coping and catastrophizing to pain-related attentional biases in women.

## Materials and Methods

### Recruitment

The study protocol followed the rules stated in the Declaration of Helsinki and was approved by the local Ethics Committee of the University Hospital Essen, Germany (approval #10-4493). Recruitment and data collection were conducted between August 2016 and December 2017. The recruitment and screening process followed our group's established procedures in place for all visceral pain studies involving rectal distensions, and included a structured telephone screening and personal interview with a medical examination conducted by a physician.

To calculate the required sample size, we performed a power analysis using G^*^Power (version 3.1.9.2) (29). To detect between-groups effects of sex, we specified two groups, i.e., women and men, and eight measurements, i.e., four conditions of the dot-probe task at baseline and during the test phase presenting CS^+^ and CS^−^ each with a neutral cue, CS^+^ and CS^−^ together and one condition only presenting neutral cues. Based on the meta-analysis by (11) reporting attentional biases toward signals of impending experimental pain in healthy individuals, we assumed a medium effect size of Cohen's *d* = 0.676 (conforming to Cohen's *f* = 0.338). With an actual power of 0.95, the required sample size included 66 participants with a critical *F*-value of 3.99.

Based on local advertisement, we were initially contacted via telephone or email by a total of *N* = 143 interested individuals. After providing more detailed study information, *N* = 102 individuals agreed to participate in our structured telephone screening. Exclusion criteria were age <18 or >45 years, body mass index (BMI) <18 or >30, any known medical or psychological health condition, chronic medication use except occasional use of over-the-counter allergy or pain medications, and prior participation in any other conditioning study conducted by our group. Moreover, we excluded free-cycling women based on self-report to avoid confounding effects of cyclical fluctuations of sex hormones. During telephone screening, a total of *N* = 26 individuals were excluded. All others (*N* = 76) were then scheduled for a personal interview at the University Hospital Essen. During the interview, participants were screened for current anxiety or depression symptoms using the German version of the Hospital Anxiety and Depression Scale (HADS; Cronbach's alpha α = 0.80 for the anxiety subscale and α = 0.81 for the depression subscale) (30) and for symptoms suggestive of any functional or organic gastrointestinal condition based on a standardized in-house questionnaire (31). All participants were further evaluated digitally for perianal tissue damage (e.g., painful hemorrhoids). Based on these screening criteria, *N* = 5 individuals were excluded due to HADS scores ≥8, *N* = 3 due to increased gastrointestinal symptoms ≥13, and *N* = 1 due to anal tissue damage. Further, on the day of the experimental study, two participants missed the appointment and one participant terminated the study during the second run of the dot-probe task. The final sample that we herein report on thus includes 64 healthy volunteers (32 women, 32 men).

### Study Design and Procedures

Prior to study participation, all participants were instructed not to eat, drink, or exercise within 2 h before arrival to the laboratory. Participants completed the study protocol within ~2 h between 09:00 and 16:00 h. Upon arrival, participants gave written informed consent and completed the questionnaire battery and then underwent the TMT and Stroop test (see below). For women, pregnancy was routinely excluded by commercially available urinary test upon arrival. All participants were tested in a medically-equipped, sound-shielded, and dimly lit room and were positioned in a hospital bed. The rectal balloon was subsequently placed, sensory thresholds were determined, and distension pressure for implementation during conditioning was individually calibrated based on the rectal pain threshold (for details, see below). All instructions and tasks were presented on a 22-inch widescreen monitor with 60 Hz refresh rate and a display resolution of 1,680 ×1,050 pixels at a viewing distance of ~140 cm. All measurements were performed by the same female tester.

**Figure 1:**
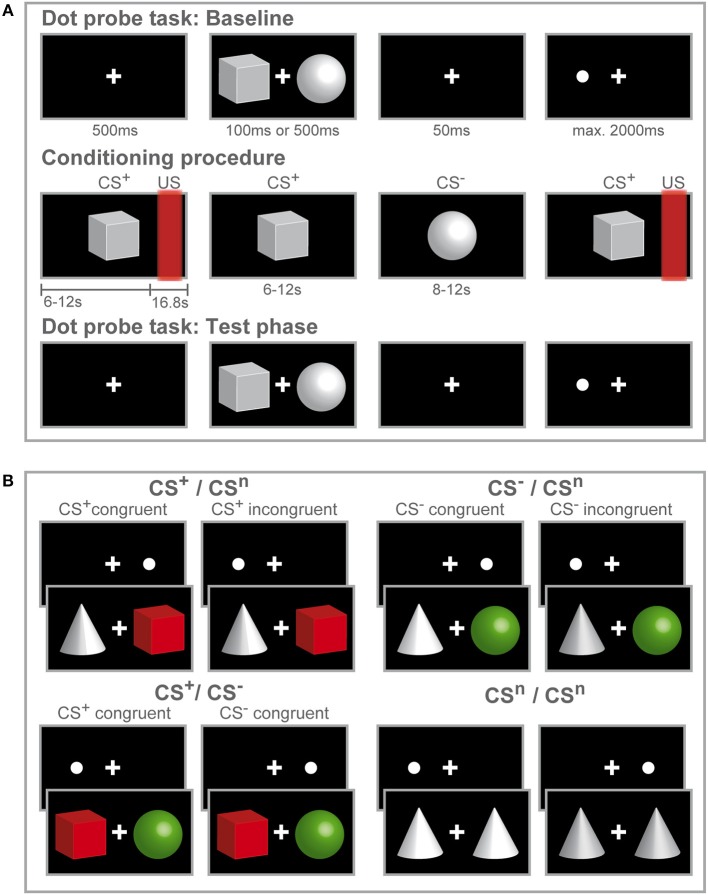
Study design and experimental procedure. Schematic illustration of the experimental procedure **(A)** and conditions during the dot-probe task **(B)**. **(A)** Participants initially accomplished a dot-probe task (baseline) during which three different visual cues were used as stimulus material. Participants were instructed to respond by button press to the dot appearing either on the left or right side of the fixation cross. During the subsequent fear-conditioning procedure, one visual cue (CS^+^) was repeatedly paired with a rectal distension (US) while a second visual cue (CS^−^) was presented without US. Only two out of three visual cues were presented during conditioning. Afterwards participants completed a second run of the dot-probe task (test phase) with the same visual stimuli presented during the baseline task. After each phase, visual analog scale (VAS) ratings of CS valence and CS-US contingency (only after fear conditioning) were accomplished. **(B)** In the dot-probe task, counterbalanced and randomized order of the three different visual cues yielded four conditions. The cues are color-coded for visual purposes only to illustrate the different CS valences acquired after conditioning. For the CS^+^ /CS^n^ and CS^−^ /CS^n^ conditions, trials were considered congruent if the dot-probe appeared at the location previously occupied by the CS^+^ (shown in red) or CS^−^ (shown in green) and incongruent, if the dot appeared at the location previously occupied by the neutral cue (shown in gray). When CS^+^ and CS^−^ where presented together, the dot-probe was considered CS^+^-congruent when it appeared at the location previously occupied by the CS^+^ and CS^−^-congruent when the dot appeared at the location previously occupied by the CS^−^. During the neutral condition, only neutral cues were presented.

The study design consisted of a mixed-group (women, men) repeated-measures design with three consecutive experimental phases (Figure 1A): A visual dot-probe task (baseline, for details on the task see below) was followed by fear conditioning, after which the same visual dot-probe task was repeated during the test phase. For conditioning, explained in detail below, we implemented an established differential delay conditioning paradigm with individually calibrated painful rectal distensions as interoceptive unconditioned stimuli, carried out with a pressure-controlled barostat system, and visual cues as conditioned stimuli (CS). The total duration of the dot-probe task varied between participants with a notional minimum duration of 4.67 min assuming overall reaction times of 200 ms and a maximum duration of 13.07 min assuming the longest possible reaction time of 2,000 ms with an average of 8.87 min. After completion of the first dot-probe task, instructions were given for the conditioning procedure that commenced immediately afterwards. The total duration of the conditioning procedure was 7.77 min. Subsequently, the study investigator informed the participant that a second run of the dot-probe task using the same procedure as during the first run will immediately start. Together, average duration for all three experimental phases was 25.51 min.

### Questionnaire Battery and Neuropsychological Testing

On the study day, participants initially completed a questionnaire battery assessing sociodemographic variables as well as several psychological characteristics. Trait anxiety was measured with the State Trait Anxiety Inventory (STAI-T; α between 0.88 and 0.94) (32) as a self-report of stable aspects of anxiety proneness. To assess situation-specific aspects of cognitive coping and catastrophizing with pain, we used the Pain-Related Self Statements Scale (PRSS; α = 0.92) (33). In addition, we measured current emotional states and fluctuations using the Positive and Negative Affect Schedule (PANAS; for both subscales α = 0.86) (34).

Moreover, the Trail Making Test (TMT) and Stroop Color Word Test were administered as control parameters for possible differences between women and men in attentional and executive functioning. The TMT is a neuropsychological task proposed to measure selective attention and processing speed in version A and set shifting in version B (35, 36), with first evidence suggesting that healthy women perform worse on version B (37). The Stroop test is thought to provide a measure of cognitive inhibition (38, 39). However, evidence regarding sex differences remains scarce and has so far yielded inconclusive results (40, 41).

### Visual Dot-Probe Task and Attentional Bias Assessment

The visual dot-probe task employed in the present study was used to assess the phenomenological characteristics of attentional biases that have previously been determined in various empirical investigations (7): attentional avoidance, engagement and disengagement. Most studies on attentional biases in chronic pain so far utilized words or varying categories of pictures or facial expressions that however raised concerns about their ecological validity and that may not be sufficiently intense to induce attentional biases (42). Therefore, it has been proposed that stimuli need to be sufficiently matched to the individual qualities of pain-related fear (26), for instance through instructed fear stimuli (43) or differential conditioning (44, 45). We herein modified the original dot-probe task (46) by utilizing differentially valenced visual stimuli induced through differential, pain-related fear conditioning.

Moreover, many studies utilizing the dot-probe task have encountered methodological and analytical problems due to the absence of baseline measures. Herein, the implementation of neutral stimuli within conditions creates a neutral baseline against which responses toward threat and safety cues can be compared and therefore allows to specifically determine the type of attentional bias observed (47, 48). Likewise, the implementation of a baseline measurement of the dot-probe task before pain-related fear conditioning allows assessing intra-individual changes in attentional biases in response to experimentally induced pain-related fear and safety cues in a within-subject design.

Finally, while common dot-probe tasks often employ static stimulus durations, we furthermore aimed to elucidate the time course of attentional biases by varying the time between stimulus onset and appearance of the probe. According to a two-stage model proposing that initial attentional vigilance is followed by attentional avoidance, some studies yet revealed that the utilization of short and long stimulus durations captures attentional features occurring prior to attentional avoidance (49, 50). Moreover, Koster et al. (8) have emphasized the potential interaction of stimulus duration and threat value, indicating that attentional biases may show different phenomenological characteristics which has been corroborated by multiple studies demonstrating stimulus duration to exert a moderating effect (8, 51, 52). Specifically, stimuli of high biological significance like pain can immediately draw attention to the potential source of threat even before conscious perception and evaluation (53), suggesting that acquired signals of imminent threat as well as safety cues signaling the absence of pain may likewise occupy different phenomenological and temporal characteristics. We herein adopted presentation times from (8) who demonstrated the impact of stimulus duration and threat value on the time course of attentional biases using pictorial stimuli.

Each trial consisted of the following sequence (Figure 1A): First, a fixation cross was displayed in the center of the screen for 500 ms. Then, a stimulus pair of geometric symbols was presented with one stimulus presented left and one stimulus presented right from the fixation cross with a variable duration of either 100 or 500 ms. After a blank screen depicting the fixation cross for 50 ms, a dot appeared either on the left or right side from the fixation cross at the same location occupied by one of the stimuli before. Participants were asked to respond as fast as possible to the dot with button press of the corresponding arrow key on a keyboard. If a response was registered, the dot disappeared or if no response was registered, the dot lasted until a maximum response time set at 2,000 ms. Inter-trial intervals (ITI) were 750 ms. Randomizing and counterbalancing stimulus presentation across trials and stimuli across the left and right side of the fixation cross resulted in four conditions (Figure 1B), presenting the CS^+^ with a neutral cue (CS^+^ + CS^n^; CS^n^ + CS^+^), presenting the CS^−^ with a neutral cue (CS^−^ + CS^n^; CS^n^ + CS^−^), conditions presenting the CS^+^ and CS^−^ together (CS^+^ + CS^−^; CS^−^ + CS^+^) and neutral trials (CS^n^ + CS^n^). For each run of the dot-probe task, 280 trials were presented, 80 per each condition presenting a CS^+^ and/ or CS^−^ and 40 trials presenting only neutral cues.

As main outcome, reaction time (RT) was recorded on each trial to assess the speed of response to the location of the dot-probe. Before statistical analyses, premature responses <100 ms, missing and false responses were removed from RT data according to empirical recommendations for analyses of the dot-probe task (50). Mean RT was calculated for each condition, type of congruency (congruent, incongruent) and stimulus duration (100, 500 ms) for the dot-probe task at baseline and during the test phase. Type of congruency was determined using target *t* and dot-probe *d* appearing either on the left *l* or right *r* side. A trial is considered congruent when the dot appears at the same location as the previously displayed target stimulus, calculating the mean RT as RT_congruent_ = (RT_tldl_ + RT_trdr_)/2. On incongruent trials, the dot appears at the location of the neutral stimulus and is calculated as RT_incongruent_ = (RT_tldr_ + RT_trdl_)/2. Trials presenting both CS^+^ and CS^−^ represent a special case where trials can be determined as CS^+^-congruent if the dot appears at the location of the CS^+^, and CS^−^-congruent if the dot appears at the location of the CS^−^. Likewise, mean RT for the neutral condition presenting only neutral CS that were not subjected to fear conditioning were calculated as RT_neutral_ = (RT_tldl_+ RT_trdr_ + RT_tldr_ + RT_trdl_)/4. Outlying RT values were then identified based on the interquartile range (IQR) with a lower threshold of Q1−1.5 × IQR and the upper threshold of Q3−1.5 × IQR.

Indices of attentional avoidance, engagement and disengagement were calculated for each condition following previous studies (8, 54). Attentional avoidance was calculated as RT_incongruent_–RT_congruent_ with negative scores indicating stronger attentional avoidance. Attentional engagement was calculated as RT_neutral_–RT_congruent_ with positive scores indicating enhanced attentional capture and negative scores indicating slower attentional engagement. Attentional disengagement was calculated as RT_neutral_–RT_incongruent_ with negative scores indicating stronger attentional vigilance with difficulty to divert onto another stimulus and positive scores indicating faster attention away from the cue. For all these indices, a score of zero implies no differences either in attentional avoidance, engagement or disengagement.

### Visceral Pain Model and Conditioning

Rectal distensions were carried out with a pressure-controlled barostat system (modified ISOBAR 3 device, G & J Electronics, Toronto, ON, Canada). Sensory and pain thresholds were determined using the ascending method of limits delivering distensions with random pressure increments of 5 mmHg and a maximal distension pressure of 50 mmHg as previously described (31, 55, 56). During conditioning, individually calibrated rectal distensions were implemented as interoceptive painful US. To this end, prior to the baseline dot-probe task, individual US intensity was carefully titrated. Participants rated selected pressures (i.e., starting with pressures just below pain threshold) on a visual analog scale (VAS) ranging from 0 to 100 with endpoints labeled “not painful at all” and “very painful.” When participants rated pain intensities between 60 and 70, the corresponding pressure was chosen for US presentation during the conditioning procedure.

During conditioning, one geometric visual symbol (CS^+^) was consistently paired with a painful rectal distension (US) while a second visual cue (CS^−^) was never followed by the US (differential delay conditioning) (Figure 1A). Overall, 32 CS were presented (16 CS^+^ and 16 CS^−^) in pseudo-randomized order with a 75% reinforcement schedule, i.e., 12 out of 16 CS^+^ were followed by a US and 4 CS^+^ remained unpaired. Duration of distensions was 16 s and US onset varied randomly between 6 and 12 s after CS^+^ onset with both stimuli co-terminating. Please note that reinforced CS^+^ were presented longer than non-reinforced CS^+^. Inter-trial intervals (ITI) were 20 s. In our previous conditioning studies, this conditioning paradigm revealed successful pain-related learning to threat and safety cues on behavioral and neural measures in healthy individuals (19, 56) and in patients with chronic visceral pain (16, 17).

### Behavioral Measures of Emotional Learning and Contingency Awareness

Online visual analog scales (VAS) were used to assess learning-induced changes in CS valence along with expected changes in cognitive aspects that together verify the efficacy of conditioning herein (57). Prior pain-related conditioning studies from our own group [reviewed in (58, 59)] and in the broader fear conditioning literature support the notion that conditioned changes in cue valence constitutes a sensitive and relevant behavioral measure (60, 61). CS valence was assessed at three time points: (1) after the first dot-probe task and prior to conditioning (baseline), (2) after conditioning and (3) following the second dot-probe task. Participants were prompted to indicate how they perceived each of the visual cues by presenting a digitized vertical 200 mm scale with end points labeled “very unpleasant” and “very pleasant,” indicating “neutral” in the middle of the scale. These values were then transformed into a scale with end points −100 indicating “very pleasant,” 100 indicating “very unpleasant” and “neutral” at 0 as previously accomplished in all of our prior conditioning work with this visceral pain model (13, 19–21). After conditioning, contingency awareness was assessed with ratings on how often each of the visual cues was followed by a rectal distension with corresponding scale end points labeled “never” (0%) and “always” (100%).

### Statistical Analyses

Analyses of all data were carried out with the Statistical Package for the Social Sciences (SPSS, IBM Corp. IBM SPSS Statistics for Windows, Version 22.0. Armonk, NY: IBM Corp.). Initially, we analyzed whether women and men were comparable with respect to social demographics, psychological state and trait variables as well as perceptual and pain thresholds. All variables were normally distributed and analyzed by means of two-sample *t*-tests.

For CS valence ratings, we conducted a repeated measures ANCOVA with within-subject factors CS (CS^+^, CS^−^, CS^n^) and phase (baseline, conditioning, test phase), between-subject factor sex (women, men), and BMI as covariate due to a significant difference between women and men for each condition separately. For CS-US contingency, paired *t*-tests were calculated with values assessed after conditioning for women and men separately.

For attentional bias indices, we carried out repeated measures ANCOVAs with the within-subject factors phase (baseline, test phase), stimulus duration (100, 500 ms), the between-subject factor sex (women, men), and BMI as covariate. To address the specificity of attentional bias to threat and safety cues, we further compared trials presenting a CS with a neutral cue with trials presenting both CS^+^ and CS^−^ using repeated measures ANCOVAs with within-subject factors condition (CS + CS^n^, CS^+^ + CS^−^), phase (baseline, test phase), the between-subject factor sex (women, men), and BMI as covariate. Results are reported for significant interactions with Greenhouse-Geisser correction and *post-hoc* testing was accomplished with Bonferroni correction to adjust for multiple comparisons. To test the effects of response slowing as a potential confound in analyses of attentional biases, we furthermore performed ANCOVAS using reaction time data. For these analyses, conditions presenting the CS^+^ with the neutral cue were compared with conditions presenting only neutral cues to test for cue (CS^+^ + CS^n^; CS^n^ + CS^n^) × congruency (congruent trials; incongruent trials) interactions.

Finally, we carried out multiple regression analyses to predict changes in indices of attentional avoidance, engagement and disengagement from baseline to test phase for each condition separately. Predictors included sex, psychological vulnerability factors of trait anxiety (STAI-T), positive and negative affect (PANAS-P, PANAS-N), cognitive coping and catastrophizing (PRSS) and CS valence changes. Moreover, interaction terms with sex were calculated by multiplying sex by each predictor. For all regression models, sex was entered into the first block and always maintained. Into the second block, a pair including the main effect and interaction with sex for one predictor was entered. If the regression model did not yield significance, the interaction term was removed first and the subsequent regression model was set up with sex in the first block and the main effect of the predictor in the second block. If the main effect did not yield significance, it was removed from the model and the subsequent regression model was set up with sex only. Results are reported with *F*- and *p*-values, adjusted R^2^ and for the coefficients, standardized beta-, *t*-, and *p*-values are given. If the main effect and/ or interaction term was found as significant predictor, subsequent regression analyses were carried out with the main effect of the predictor for women and men separately, reporting *F*- and *p*-values and adjusted R^2^ for the model and for the coefficient the beta- and *t*-values. Detailed results of the regression analyses are given in the Supplementary Material.

All data are given as mean ± standard error of the mean (SEM), unless indicated otherwise.

## Results

### Sample Characteristics

In total, 64 healthy volunteers (32 women, 32 men) completed the study. Sociodemographic and psychological characteristics were comparable between men and women, except for a lower BMI in women (Table 1). Positive and negative affective states (PANAS), trait anxiety (STAI-T) and pain-related cognitions including catastrophizing and active coping (PRSS), all assessed upon arrival to the laboratory on the study day, did not reveal differences between women and men. Likewise, neuropsychological attention tests including the TMT and Stroop task as well as rectal and pain thresholds were comparable between groups (Table 1).

**Table 1:** Sample characterization.

	**Full sample**	**Women**	**Men**	**Statistics**
**(A) Measured during screening**				
Age	28.22 ± 1.00	28.53 ± 1.71	27.91 ± 1.06	*t* = 0.31, *p* = .757
Body mass index	22.79 ± 0.33	21.92 ± 0.41	23.66 ± 0.47	*t* = 2.80, *p* = .007
Gastrointestinal symptoms	2.70 ± 0.36	3.13 ± 0.55	2.28 ± 0.46	*t* = 1.18, *p* = .241
HADS anxiety	3.70 ± 0.32	4.16 ± 0.48	3.25 ± 0.42	*t* = 1.44, *p* = .156
HADS depression	1.76 ± 0.28	1.29 ± 0.26	2.22 ± 0.48	*t* = 1.68, *p* = .096
**(B) Measured on study day**				
STAI Trait	35.67 ± 0.89	36.75 ± 1.35	34.59 ± 1.16	*t* = 1.21, *p* = .230
PANAS positive	3.25 ± 0.08	3.27 ± 0.12	3.24 ± 0.11	*t* = 0.15, *p* = .885
PANAS negative	1.39 ± 0.06	1.41 ± 0.09	1.37 ± 0.08	*t* = 0.37, *p* = .715
PRSS catastrophizing	0.80 ± 0.10	0.77 ± 0.17	0.83 ± 0.12	*t* = 0.31, *p* = .762
PRSS coping	3.54 ± 0.10	3.57 ± 0.14	3.52 ± 0.15	*t* = 0.24, *p* = .811
TMT version A	26.30 ± 1.03	26.59 ± 1.60	26.00 ± 1.33	*t* = 0.29, *p* = .776
TMT version B	53.70 ± 2.36	54.72 ± 2.34	52.69 ± 4.13	*t* = 0.43, *p* = .670
SCWT word reading	30.65 ± 0.64	30.97 ± 1.09	30.34 ± 0.69	*t* = 0.49, *p* = .628
SCWT color naming	47.29 ± 1.10	48.32 ± 1.72	46.28 ± 1.39	*t* = 0.93, *p* = .358
SCWT color-word naming	69.25 ± 1.66	70.61 ± 2.68	67.94 ± 2.00	*t* = 0.80, *p* = .425
Perception threshold	15.75 ± 0.82	16.33 ± 1.22	15.17 ± 1.11	*t* = 0.71, *p* = .483
Pain threshold	39.21 ± 1.29	38.04 ± 1.90	40.34 ± 1.75	*t* = 0.90, *p* = .375

*Overview of sociodemographic and psychological variables as well neuropsychological attention test results obtained during screening procedure (A) and on study day (B). Psychological variables were assessed with the Hospital Anxiety and Depression Scale (HADS), State-Trait Anxiety Inventory (STAI) including only the trait subscale, Positive, and Negative Affective Schedule (PANAS) including positive and negative subscales and Pain-Related Self Statements Scale (PRSS) including subscales of pain catastrophizing and coping. Neuropsychological attention tests included the Trail Making Test (TMT) with versions A and B as well as the Stroop color word test (Stroop) with conditions word reading, color naming and the interference condition of color-word naming, all results given in seconds. During the individual rectal distention calibration procedure, perception, and pain thresholds were measured in mmHG. All values are given as mean ± standard error of the mean (M ± SEM). Differences between women and men were calculated by means of independent sample t-tests*.

### CS Valence and CS-US Contingency

**Figure 2:**
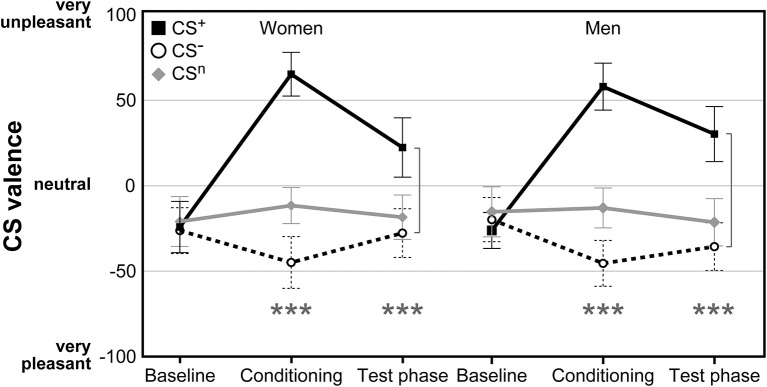
Ratings of CS valence assessed on visual analog scales (VAS) before and after conditioning as well as after the test phase. Both women and men demonstrated significant increases in CS^+^ aversiveness and increases in CS^−^ pleasantness following acquisition without differences between groups. ***p < 0.001.

For CS valence (Figure 2; for single subject data see Figure S1), analysis revealed a significant time × CS interaction (*F* = 2.79, *p* = .038, η^2^ = .05), supporting the efficacy of the conditioning procedure without any effect of sex (*F* = 0.01, *p* = .943). While ratings of all CS were expectedly comparable and neutral at baseline (all *p* > .598), after conditioning both women and men perceived the CS^+^ as significantly more unpleasant and the CS^−^ as more pleasant compared to the neutral cue (both *p* < .001). The difference between CS^+^ and CS^−^ was still evident after the test phase (*p* < .001), indicating persisting effects of conditioning. Likewise, both men and women were similarly aware of conditioning contingencies for both CS^+^ and CS^−^, as supported by changes in CS-US contingency ratings assessed after conditioning with higher perceived contingency for CS^+^-US pairings compared to CS^−^-US pairings in women (CS^+^-US contingency M ± SEM: 77.41% ± 2.46; CS^−^-US contingency: 15.69% ± 3.63; *t* = 11.00, *p* < .001), and men (CS^+^-US contingency: 81.63% ± 2.18; CS^−^-US contingency: 11.41% ± 3.46; *t* = 15.42, *p* < .001).

### Sex Differences in Indices of Attentional Bias

#### Attentional Avoidance

For attentional avoidance indices (Table 2; for single subject data see Figure S2), analyses yielded no significant interactions between phase and sex (all *F* < 0.45, all *p* > .507), and no significant interactions between phase, sex and stimulus duration (all *F* < 0.53, all *p* > .470), indicating no differences in attentional avoidance between women and men.

**Table 2:** Indices of attentional avoidance.

	**Women**		**Men**					
	**Baseline**	**Test phase**	**Baseline**	**Test phase**	**Interaction effect**	***F***	***p***	***η^*2*^***
**CS**^**+**^**/CS**^**n**^	2.73 ± 2.20	5.55 ± 2.96	3.28 ± 3.67	2.88 ± 2.59	Phase × sex	0.13	.717	.00
					Phase × duration × sex	0.53	.470	.01
**CS**^**−**^**/CS**^**n**^	5.99 ± 3.53	1.66 ± 3.51	10.87 ± 2.98	5.96 ± 2.71	Phase × sex	0.45	.507	.01
					Phase × duration × sex	0.16	.691	.00
**CS**^**+**^**/CS**^**−**^	−5.89 ± 3.34	−2.78 ± 3.10	−6.72 ± 3.40	−6.17 ± 3.50	Phase × sex	0.18	.671	.00
					Phase × duration × sex	0.38	.542	.01

*Descriptive statistics and results from ANCOVAs comparing indices of attentional avoidance across phase (baseline vs. test phase), stimulus duration (100 ms, 500 ms) and sex (women vs. men). Conditions include presentation of CS^+^ and CS^−^ each with a neutral cue and presentation of CS^+^ and CS^−^ together. Descriptive statistics are given as mean values across 100 and 500 ms stimulus duration ± standard error of the mean (M ± SEM)*.

#### Attentional Engagement

**Figure 3:**
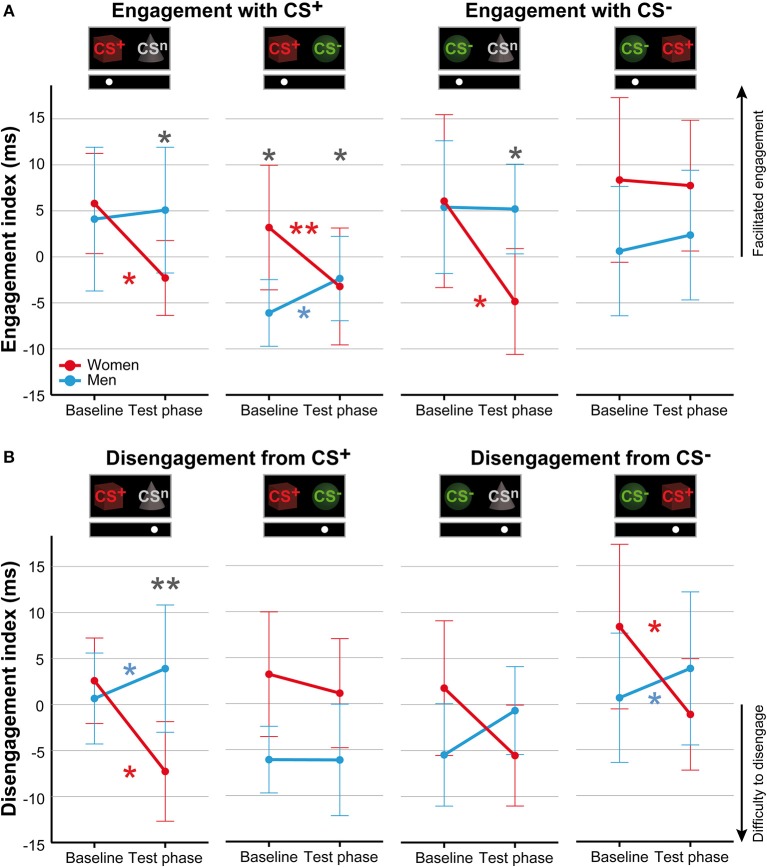
Sex differences in attentional biases. Mean indices of attentional engagement **(A)** and disengagement **(B)** given in ms for women (red) and men (blue) for the dot-probe tasks accomplished at baseline and during the test phase. Please note that mean values depicted in the figure are not corrected for BMI that was used as covariate in statistical analyses. Significant differences between women and men are given with gray asterisks and within-group differences between baseline and test phase are given with the corresponding color code for women and men. Men compared to women showed higher attentional engagement with the CS^+^ and CS^−^ when presented with the neutral cue and with the CS^+^ when presented together with the CS^−^. Women compared to men demonstrated stronger difficulties to disengage attention from the CS^+^ when presented with the neutral cue. **p < .010; *p < .050.

For attentional engagement indices (Table 3, Figure 3A; for single subject data see Figure S3), analyses revealed significant phase × sex interactions for both conditions presenting the CS^+^ and the CS^−^ with the neutral cue (all *F* > 7.06, all *p* < .011, all η^2^ > .14). However, there was no effect of stimulus duration (all *F* < 0.61, all *p* > .441). *Post-hoc* comparisons revealed during the test phase significantly higher indices for men (all *p* < .038), indicating that men showed higher attentional engagement after fear conditioning compared to women. In contrast, women revealed significant decreases from baseline to test phase in attentional engagement indices across these conditions (all *p* < .021), while men revealed a significant increase in response to the CS^+^ when presented together with the CS^−^ (*p* = .032).

**Table 3:** Indices of attentional engagement.

	**Women**		**Men**					
	**Baseline**	**Test phase**	**Baseline**	**Test phase**	**Interaction effect**	***F***	***p***	***η^*2*^***
**CS**^**+**^**/CS**^**n**^	5.39 ± 2.62	−1.43 ± 2.19	3.93 ± 3.87	4.90 ± 3.39	Phase × sex	**9.20**	**.004**	**.16**
					Phase × duration × sex	0.12	.731	.00
**CS**^**+**^**/CS**^**−**^	3.49 ± 3.30	−3.47 ± 3.09	−6.10 ± 1.81	−2.36 ± 2.29	Phase × sex	**11.73**	**.001**	**.18**
					Phase × duration × sex	0.36	.551	.01
**CS**^**−**^**/CS**^**n**^	6.48 ± 4.57	−3.88 ± 2.95	5.40 ± 3.61	5.20 ± 2.43	Phase × sex	**7.06**	**.011**	**.14**
					Phase × duration × sex	0.03	.869	.00
**CS**^**−**^**/CS**^**+**^	9.04 ± 4.39	8.89 ± 3.63	0.62 ± 3.51	2.37 ± 3.52	Phase × sex	2.13	.151	.05
					Phase × duration × sex	0.61	.441	.01

*Descriptive statistics and results from ANCOVAs comparing indices of attentional engagement across phase (baseline vs. test phase), stimulus duration (100, 500 ms) and sex (women vs. men). Conditions include presentation of CS^+^ and CS^−^ each with a neutral cue and presentation of CS^+^ and CS^−^ together. The underscore indicates the appearance of the dot-probe at the location either of the CS^+^ or CS^−^. Descriptive statistics are given as mean values across 100 and 500 ms stimulus duration ± standard error of the mean (M ± SEM). Significant interactions are given in bold*.

To further investigate the specificity of attentional engagement, conditions presenting one CS with the neutral cue were compared with conditions presenting both the CS^+^ and CS^−^. For the CS^−^, analyses revealed an interaction between condition, phase and sex that marginally missed to yield significance (*F* = 3.87, *p* = .054). Exploratory *post-hoc* analyses revealed for women significantly higher attentional engagement with the CS^−^ when presented with the CS^+^ as compared to the concomitant presentation of the neutral cue (*p* = .012) while for men no effects were observed.

#### Attentional Disengagement

For disengagement indices (Table 4, Figure 3B; for single subject data see Figure S4), analyses revealed significant phase × sex interactions for conditions when the CS^+^ and CS^−^ were each presented with the neutral cue as well as for the CS^−^ when presented with the CS^+^ (all *F* > 6.44, all *p* < .014, all η^2^ > .11). Stimulus duration again had no effect (all *F* < 0.66, all *p* > .421). *Post-hoc* comparisons between women and men during the test phase yielded significance only when the CS^+^ was presented with the neutral cue (*p* = .009) indicating that women had stronger difficulties to disengage attention from the CS^+^. For the remaining conditions, results did not survive Bonferroni correction (CS^−^/CS^n^ *p* = .080; CS^−^/CS^+^ *p* = .062). Moreover, comparisons between baseline and test phase revealed that women had stronger difficulties to disengage from the CS^+^ when presented with the neutral cue (*p* = .010) and from the CS^−^ when presented with the CS^+^ (*p* = .023) while men demonstrated significant increases in these conditions (both *p* < .039). Further analyses on the specificity of attentional disengagement between conditions did not yield significance.

**Table 4:** Indices of attentional disengagement.

	**Women**		**Men**					
	**Baseline**	**Test phase**	**Baseline**	**Test phase**	**Interaction effect**	***F***	***p***	***η^*2*^***
**CS**^**+**^**/****CS**^**n**^	2.47 ± 2.25	−6.98 ± 2.64	0.65 ± 2.47	3.90 ± 3.46	Phase × sex	**10.36**	**.002**	**.18**
					Phase × duration × sex	0.66	.421	.01
**CS**^**+**^**/****CS**^**−**^	9.04 ± 4.39	−0.69 ± 2.98	0.62 ± 3.51	3.81 ± 4.16	Phase × sex	1.92	.173	.04
					Phase × duration × sex	0.47	.497	.01
**CS**^**−**^**/****CS**^**n**^	2.21 ± 3.57	−5.54 ± 2.66	−5.48 ± 2.78	−0.67 ± 2.39	Phase × sex	**6.44**	**.014**	**.11**
					Phase × duration × sex	0.05	.831	.00
**CS**^**−**^**/****CS**^**+**^	3.49 ± 3.30	1.31 ± 2.88	−6.10 ± 1.81	−6.14 ± 3.03	Phase × sex	**9.73**	**.003**	**.15**
					Phase × duration × sex	0.06	.802	.00

*Descriptive statistics and results from ANCOVAs comparing indices of attentional disengagement across phase (baseline vs. test phase), stimulus duration (100, 500 ms) and sex (women vs. men). Conditions include presentation of CS^+^ and CS^−^ each with a neutral cue and presentation of CS^+^ and CS^−^ together. The underscore indicates the appearance of the dot-probe at the location either of the CS^+^ or CS^−^. Descriptive statistics are given as mean values across 100 and 500 ms stimulus duration ± standard error of the mean (M ± SEM). Significant interactions are given in bold*.

#### Response Slowing

Previous research on the allocation of visuospatial attention demonstrated a response slowing effect of threat indicating that differences in reaction times between trials containing a threat cue and trials containing a neutral cue might arise from avoidance or freezing of motor responses [e.g., (57, 58)]. We tested for this effect by comparing reaction times from conditions presenting the CS^+^ with a neutral cue with conditions presenting the neutral cue only for 100 and 500 ms stimulus duration. For 100 ms stimulus duration, the cue x congruency interaction yielded significance (*F* = 7.80, *p* = .007, η^2^ = .14) with *post-hoc* comparisons demonstrating higher reaction times for incongruent compared to congruent CS^+^ trials (*p* = .017). However, there were no significant differences between the CS^+^ and the neutral condition for either congruent or incongruent trials (both *p* > .102). For 500 ms stimulus duration, there was no significant cue x congruency interaction (*F* = 1.81, *p* = .184) indicating no effect of response slowing.

### Sex-Specific Associations Between Emotional Learning, Psychological Traits and Attentional Biases

Multiple regression analyses with the main effects of sex, psychological vulnerability factors, and changes in CS valence as well as the interaction terms considering sex were calculated to predict changes in attentional biases from baseline to test phase.

For attentional avoidance, the model with the interaction between sex and negative affect as well as the interaction between sex and active coping yielded significance (*F* = 4.48, *p* = .007, adj. *R*^2^ = .14). Both, the interaction sex^*^negative affect (β = 0.48, *t* = 3.04, *p* = .004) and sex^*^active coping emerged as significant predictors (β = −0.45, *t* = 2.26, *p* = .028) while sex alone did not yield significance (β = 0.06, *t* = 0.26, *p* = .797) (for more details, see Table S1). Subsequent analyses revealed a larger slope for men (*F* = 9.03, *p* = .005, adj. *R*^2^ = .23, β = 0.48, *t* = 3.01) compared to women (*F* = 0.02, *p* = .879, adj. *R*^2^ = −.03, β = 0.03, *t* = 0.15), indicating that only in men higher negative affect was associated with stronger avoidance of the CS^+^ when presented with the CS^−^. However, for active coping neither regression model yielded significance although the slope observed for men (*F* = 2.76, *p* = .107, adj. *R*^2^ = .06, β = −0.29, *t* = 1.67) was higher compared to women (*F* = 2.76, *p* = .107, adj. *R*^2^ = .06, β = −0.29, *t* = 1.67).

For attentional engagement, sex was found as a significant predictor (*F* = 4.27, *p* = .043, adj. *R*^2^ = .05, β = 0.25, *t* = 2.07) for the condition presenting the CS^+^ together with the CS^−^ while regression models including psychological vulnerability factors and changes in CS valence failed to reach significance (*F* = 3.77, *p* = .057, adj. *R*^2^ = .04) (Table S2).

For attentional disengagement, sex emerged as a significant predictor for conditions presenting the CS^+^ with the neutral cue (*F* = 4.95, *p* = .030, adj. *R*^2^ = .06, β = 0.27, *t* = 2.22) and the CS^−^ with the neutral cue (*F* = 5.14, *p* = .027, adj. *R*^2^ = .06, β = 0.28, *t* = 2.27) (Tables S3, S4). Moreover, for the condition presenting the CS^−^ together with the CS^+^, the model including sex, negative affect and sex^*^negative affect yielded significance (*F* = 4.93, *p* = .004, adj. *R*^2^ = .16) (Table S5). Herein, sex (β = 1.13, *t* = 3.18, *p* = .002), negative affect (β = 0.84, *t* = 2.36, *p* = .022) and the interaction between sex and negative affect (β = 1.54, *t* = 3.19, *p* = .002) were found as significant predictors. Subsequent regression analyses for the interaction effect revealed a larger slope for men (*F* = 16.99, *p* < .001, adj. *R*^2^ = .34, β = −0.60, *t* = 4.12) compared to women (*F* = 0.36, *p* = .555, adj. *R*^2^ = −.02, β = 0.11, *t* = 0.60), indicating that only in men higher negative affect was related to stronger difficulties to disengage from the CS^−^.

## Discussion

Although the broad role of fear and hypervigilance in the transition from acute to chronic pain is widely acknowledged, pain-related attentional biases as putative neurocognitive mechanism remain incompletely understood. In addition, sex differences as they are considered highly relevant to both acute and chronic pain are rarely systematically studied, especially in the context of visceral pain (62). The aim of this study was to elucidate attentional biases induced by pain-related conditioning in healthy women and men. To this end, we implemented a visual dot-probe task before and after differential fear conditioning with visceral pain as highly salient and clinically relevant interoceptive US (12, 13, 63). Conditioning successfully induced emotional pain-related learning to threat and safety cues, as evidenced by differential changes in cue valence. Consistent with behavioral and neural findings in our earlier conditioning studies (12, 19, 21), visual cues that were contingently paired with visceral pain became highly unpleasant predictors of threat, likely reflecting conditioned pain-related fear in anticipation of visceral pain. In contrast, cues that predicted the absence of impending pain acquired positive emotional valence, consistent with their role as conditioned safety signals. There was no evidence of sex differences in emotional pain-related learning, confirming earlier behavioral results in a different, smaller sample of healthy volunteers (23). Similarly, men and women did not differ in contingency awareness of cue-pain relationships, which was fairly accurate in both sexes. Interestingly, comparing men and women with respect to attentional avoidance, facilitated engagement, and difficulties to disengage from visceral pain-related threat and safety cues revealed novel evidence supporting sex differences in attentional biases. Specifically, men showed more facilitated attentional engagement with both threat and safety cues. Women, on the other hand, demonstrated more pronounced difficulties to disengage from threat in the presence of a neutral cue. However, when both threat and safety cues were presented, women showed stronger difficulties to disengage from the safety cue. While these findings do not support our hypothesis of facilitated threat engagement in women, they are consistent with a proposed bias toward safety cues. While the role of conditioned safety signals remains incompletely understood and arguably underappreciated, we previously reported distinctly altered neural processes during safety learning in healthy women (18) as well as in women with IBS (17). Specifically, IBS patients demonstrated a more pronounced positive valence increase to conditioned safety cues, higher awareness of safety cue contingencies, as well as greater neural responses involving regions relevant to reward processing (17) and conditioned autonomic, somatomotor and cognitive fear responses (16). Distinct neural networks engaged during the acquisition and extinction of conditioned threat vs. safety cues have not only been reported in our model, but also more broadly in the fear conditioning literature (64). Together, our findings support that visceral pain-related fear conditioning induces attentional biases differently in healthy women and men, supporting a role of sex or gender in attentional mechanisms underlying hypervigilance.

Attentional biases to signals predicting experimental pain have previously been shown, albeit outside of interoceptive, visceral pain, as summarized in recent meta-analyses (11, 65). Our results complement results gathered in different conditioning paradigms (47, 48, 66–69), and extend knowledge regarding the role of pain-related fear as a putative mediator. Moreover, several psychological state and trait factors demonstrably contribute to inter-individual differences in the modulation of pain (22, 70, 71), which have thus far rarely been considered in experimental studies on pain-related attentional biases. Therefore, we explored sex differences in relationships between attentional biases and positive and negative affect, trait anxiety, pain-related coping and catastrophizing using regression analyses. Our results revealed an influence of sex on negative affect in predicting attentional threat avoidance and difficulties to disengage from the safety cue. This finding is well in line with the fear avoidance model of chronic pain, emphasizing that a higher propensity toward experiencing negative emotions impacts upon pain control and increases pain-related fear, thereby promoting avoidance behaviors (72). Interestingly, these effects were observed in men while no relationships between psychological vulnerability factors and attentional biases were observed in women. While this is at odds with our hypothesis, we cannot with certainty exclude a self-selection bias for participation due to the research setting at a university hospital and the nature of the study, specifically concerning the application of painful stimuli (73). This may have resulted in an overall sample of healthy individuals presenting with rather low anxiety and negative affect and may have specifically discouraged healthy women with higher experience of gastrointestinal symptoms. Therefore, the translation to the general population or patient populations is limited. Moreover, it has previously been suggested that individuals with high fear of pain exhibit a selective attention bias toward pain-related information, supporting that biased attentional processes mediate increased susceptibility to negative pain experience (74, 75). Our conditioning model experimentally induces fear as a state, rather than a trait assessed by questionnaire. This calls attention to the fact that fear of pain and by inference pain-related attentional biases are likely shaped by more permanent, trait-like factors, as well as more state-like learning processes regarding threat and reward. Hence, inter-individual differences in pain-related attentional biases could be explained by pre-existing differences in fear of pain, as previously shown in our visceral pain model (12), as well as by sex differences, as suggested herein.

Herein, men and women were comparable with respect to pain thresholds and pain ratings, and the intensity of pain stimuli implemented during conditioning was individually calibrated, supporting that sex differences in attentional biases were not attributable to differences in the response to pain or in the strength of emotional learning to either threat or safety cues. Further strengths of this study include the ecological validity and translational qualities of the visceral pain model, as rectal distension-induced pain is highly salient even in healthy individuals (12) and closely resembles clinical pain and related symptoms such as urgency (73) in functional gastrointestinal disorders like IBS (76). In the context of attentional bias research, the utilization of semantic and pictorial threat stimuli has raised concerns about their ecological validity and generalizability (26), resulting in calls for research with paradigms that closely resemble real-life situations (77) or consider motivational context (69). In line with these efforts, our model offers research perspectives in patient populations, especially those with somatic symptom disorder or chronic visceral pain such as in IBS. Utilizing modified Stroop and exogenous cueing tasks, IBS patients revealed alterations in attentional processing for pain and symptom-related words (78, 79) as well as situational threat words (80). This attentional bias was moreover associated with symptom severity, illness behaviors and anxiety (49, 80, 81). Hence, increased attention to interoceptive, visceral sensations may lead to the exacerbation of symptoms and distress (82) in line with the fear avoidance model, which has yet to be more fully tested in the context of chronic visceral pain and the gut-brain axis. Neuroimaging studies support this assumption by demonstrating increased functional connectivity in the salience network in IBS patients during resting state (83), rectal stimulation (84) and contextual threat situations (85). Initial support that the behavioral modification of attentional bias may improve attentional functioning and regulation of brain mechanisms related to anxiety and attention in IBS patients (86, 87), provides a treatment perspective to complement more basic mechanistic research.

Lastly, our findings are relevant toward further elucidating the neurocognitive and emotional mechanisms of associative learning and memory processes in the context of visceral pain. Brain imaging studies revealed conditioning-induced changes in the brain emotional arousal and salience networks, supporting that conditioning processes contribute to hypervigilance, possibly by engaging nocebo mechanisms (58). It has also been proposed that pain-related conditioning may impair perceptual discrimination acuity (88), enhance fear generalization (89) or interfere with normal habituation processes (90). Although our attempts to show hyperalgesia as a result of conditioning provided negative results (21, 63), data from other groups do support sensitization (91, 92) and lowered pain thresholds (93). Regardless of these inconsistencies and a clear need for further study, it is important to emphasize that different yet intricately intertwined mechanisms engaged during associative learning are clearly not mutually exclusive. They may indeed play distinct roles in modulating responses to acute pain, shaping the transition from acute to chronic pain and the maintenance of pain. Importantly, attentional biases arguably play a role in many if not all of these proposed processes, and could thus be viewed as a fundamental neurocognitive mechanism that is shaped by pain-related learning and memory processes (48, 94). This is supported by first evidence that attentional bias to threat signals is still present after extinction (47) and re-emerges during reinstatement (48), consistent with our brain imaging work on the reactivation of previously extinguished responses to conditioned pain and safety cues induced by reinstatement (20, 56) or renewal (19).

## Data Availability Statement

The raw data supporting the conclusions of this article will be made available by the authors, without undue reservation, to any qualified researcher.

## Ethics Statement

The studies involving human participants were reviewed and approved by the Ethics Committee of the University Hospital Essen, Germany. The patients/participants provided their written informed consent to participate in this study.

## Author Contributions

FL and SE planned the study, contributed to data analysis and interpretation and wrote the manuscript. FL and SK-R were involved in conducting the study. All authors: revision of the manuscript for critical intellectual content and approval of the final draft submitted. All authors contributed to manuscript revision, read and approved the submitted version.

### Conflict of Interest

The authors declare that the research was conducted in the absence of any commercial or financial relationships that could be construed as a potential conflict of interest.
