# Supplementary material for: Sex Differences Linking Pain-Related Fear and Interoceptive Hypervigilance: Attentional Biases to Conditioned Threat and Safety Signals in a Visceral Pain Model
Source: Front Psychiatry. 2020 Mar 24;11:197. doi: 10.3389/fpsyt.2020.00197 (PMC7105724; doi:10.3389/fpsyt.2020.00197)
Supplement: Supplementary file 1 [file Data_Sheet_1.docx]

Supplementary Material

# Supplementary Tables

## Table S1

| **δ Attentional avoidance CS^+^/ CS^-^** | | | | | | | | | | |
| --- | --- | --- | --- | --- | --- | --- | --- | --- | --- | --- |
|  | **Full sample** | | | | **Women** | | | **Men** | | |
|  | **β** | **t** | **p** | **tol.** | **β** | **t** | **p** | **β** | **t** | **p** |
| Sex | 0.43 | 0.13 | .896 | 1.00 |  |  |  |  |  |  |
| Trait anxiety | 0.07 | 0.53 | .598 | 0.98 |  |  |  |  |  |  |
| Sex x Trait anxiety | 0.29 | 1.23 | .222 | 0.30 |  |  |  |  |  |  |
| Positive affect | -0.07 | 0.57 | .573 | 1.00 |  |  |  |  |  |  |
| Sex x Positive affect | -0.26 | 1.07 | .287 | 0.28 |  |  |  |  |  |  |
| Negative affect | -0.50 | 1.36 | .180 | 0.10 |  |  |  |  |  |  |
| **Sex x Negative affect** | **0.48** | **3.04** | **.004** | **0.55** | 0.03 | 0.15 | .879 | 0.48 | 3.01 | .005 |
| Catastrophizing | 0.02 | 0.16 | .874 | 0.99 |  |  |  |  |  |  |
| Sex x Catastrophizing | 0.07 | 0.54 | .590 | 0.85 |  |  |  |  |  |  |
| Active coping | 0.11 | 0.29 | .770 | 0.10 |  |  |  |  |  |  |
| **Sex x Active coping** | **-0.45** | **2.26** | **.028** | **0.35** | -0.13 | 0.72 | .480 | -0.29 | 1.66 | .107 |
| δ Valence CS^+^ | 0.04 | 0.32 | .752 | 1.00 |  |  |  |  |  |  |
| Sex x δ Valence CS^+^ | 0.03 | 0.21 | .832 | 0.79 |  |  |  |  |  |  |
| δ Valence CS^-^ | -0.03 | 0.26 | .799 | 0.99 |  |  |  |  |  |  |
| Sex x δ Valence CS^-^ | -0.00 | 0.04 | .973 | 0.93 |  |  |  |  |  |  |

Results from regression analysis for attentional avoidance of the CS^+^ when presented with the CS^-^. The underscore indicates the appearance of the dot-probe. Significant predictors are indicated by bold font and are given with standardized beta coefficients. Excluded variables are given with unstandardized beta coefficients. For all predictor variables, tolerance (tol.) values from multicollinearity tests are given. For significant interaction terms with sex, results from post-hoc regression analyses are given for women and men separately.

## Table S2

| **δ Attentional engagement CS^+^/ CS^-^** | | | | | | | | | | |
| --- | --- | --- | --- | --- | --- | --- | --- | --- | --- | --- |
|  | **Full sample** | | | | **Women** | | | **Men** | | |
|  | **β** | **t** | **p** | **tol.** | **β** | **t** | **p** | **β** | **t** | **p** |
| **Sex** | **0.25** | **2.07** | **.043** | **1.00** |  |  |  |  |  |  |
| Trait anxiety | 0.15 | 1.16 | .250 | 0.98 |  |  |  |  |  |  |
| Sex x Trait anxiety | 0.13 | 0.55 | .586 | 0.30 |  |  |  |  |  |  |
| Positive affect | -0.17 | 1.39 | .169 | 1.00 |  |  |  |  |  |  |
| Sex x Positive affect | -0.28 | 1.17 | .245 | 0.28 |  |  |  |  |  |  |
| Negative affect | 0.10 | 0.77 | .442 | 1.00 |  |  |  |  |  |  |
| Sex x Negative affect | 0.14 | 0.83 | .408 | 0.55 |  |  |  |  |  |  |
| Catastrophizing | -0.17 | 1.36 | .180 | 1.00 |  |  |  |  |  |  |
| Sex x Catastrophizing | -0.15 | 1.10 | .277 | 0.87 |  |  |  |  |  |  |
| Active coping | 0.23 | 1.90 | .063 | 1.00 |  |  |  |  |  |  |
| Sex x Active coping | 0.31 | 1.51 | .137 | 0.35 |  |  |  |  |  |  |
| δ Valence CS^+^ | 0.12 | 1.01 | .319 | 1.00 |  |  |  |  |  |  |
| Sex x δ Valence CS^+^ | 0.12 | 0.86 | .392 | 0.80 |  |  |  |  |  |  |
| δ Valence CS^-^ | 0.07 | 0.54 | .592 | 0.99 |  |  |  |  |  |  |
| Sex x δ Valence CS^-^ | 0.04 | 0.33 | .741 | 0.94 |  |  |  |  |  |  |

Results from regression analysis for attentional engagement with the CS^+^ when presented with the CS^-^. The underscore indicates the appearance of the dot-probe. Significant predictors are indicated by bold font and are given with standardized beta coefficients. Excluded variables are given with unstandardized beta coefficients. For all predictor variables, tolerance (tol.) values from multicollinearity tests are given. For significant interaction terms with sex, results from post-hoc regression analyses are given for women and men separately.

## Table S3

| **δ Attentional disengagement CS^+^/ CS^n^** | | | | | | | | | | |
| --- | --- | --- | --- | --- | --- | --- | --- | --- | --- | --- |
|  | **Full sample** | | | | **Women** | | | **Men** | | |
|  | **β** | **t** | **p** | **tol.** | **β** | **t** | **p** | **β** | **t** | **p** |
| **Sex** | **0.27** | **2.22** | **.030** | **1.00** |  |  |  |  |  |  |
| Trait anxiety | 0.05 | 0.36 | .718 | 0.98 |  |  |  |  |  |  |
| Sex x Trait anxiety | 0.08 | 0.35 | .727 | 0.30 |  |  |  |  |  |  |
| Positive affect | -0.06 | 0.50 | .617 | 1.00 |  |  |  |  |  |  |
| Sex x Positive affect | -0.13 | 0.54 | .592 | 0.28 |  |  |  |  |  |  |
| Negative affect | 0.00 | 0.02 | .986 | 1.00 |  |  |  |  |  |  |
| Sex x Negative affect | 0.02 | 0.14 | .889 | 0.55 |  |  |  |  |  |  |
| Catastrophizing | -0.03 | 0.21 | .834 | 1.00 |  |  |  |  |  |  |
| Sex x Catastrophizing | 0.02 | 0.11 | .912 | 0.87 |  |  |  |  |  |  |
| Active coping | 0.19 | 1.61 | .113 | 1.00 |  |  |  |  |  |  |
| Sex x Active coping | 0.37 | 1.82 | .073 | 0.35 |  |  |  |  |  |  |
| δ Valence CS^+^ | 0.04 | 0.35 | .725 | 1.00 |  |  |  |  |  |  |
| Sex x δ Valence CS^+^ | 0.07 | 0.49 | .624 | 0.80 |  |  |  |  |  |  |
| δ Valence CS^-^ | -0.07 | 0.60 | .549 | 0.99 |  |  |  |  |  |  |
| Sex x δ Valence CS^-^ | -0.09 | 0.73 | .467 | 0.94 |  |  |  |  |  |  |

Results from regression analysis for attentional disengagement from the CS^+^ when presented with the neutral cue. The underscore indicates the appearance of the dot-probe. Significant predictors are indicated by bold font and are given with standardized beta coefficients. Excluded variables are given with unstandardized beta coefficients. For all predictor variables, tolerance (tol.) values from multicollinearity tests are given. For significant interaction terms with sex, results from post-hoc regression analyses are given for women and men separately.

## Table S4

| **δ Attentional disengagement CS^-^/ CS^n^** | | | | | | | | | | |
| --- | --- | --- | --- | --- | --- | --- | --- | --- | --- | --- |
|  | **Full sample** | | | | **Women** | | | **Men** | | |
|  | **β** | **t** | **p** | **tol.** | **β** | **t** | **p** | **β** | **t** | **p** |
| **Sex** | **0.28** | **2.27** | **.027** | **1.00** |  |  |  |  |  |  |
| Trait anxiety | 0.21 | 1.71 | .092 | 0.98 |  |  |  |  |  |  |
| Sex x Trait anxiety | 0.26 | 1.14 | .258 | 0.30 |  |  |  |  |  |  |
| Positive affect | -0.06 | 0.47 | .644 | 1.00 |  |  |  |  |  |  |
| Sex x Positive affect | -0.16 | 0.67 | .509 | 0.28 |  |  |  |  |  |  |
| Negative affect | 0.05 | 0.41 | .686 | 1.00 |  |  |  |  |  |  |
| Sex x Negative affect | 0.02 | 0.11 | .913 | 0.55 |  |  |  |  |  |  |
| Catastrophizing | 0.02 | 0.19 | .849 | 1.00 |  |  |  |  |  |  |
| Sex x Catastrophizing | 0.03 | 0.21 | .836 | 0.87 |  |  |  |  |  |  |
| Active coping | 0.09 | 0.72 | .478 | 1.00 |  |  |  |  |  |  |
| Sex x Active coping | 0.18 | 0.84 | .402 | 0.35 |  |  |  |  |  |  |
| δ Valence CS^+^ | 0.11 | 0.86 | .395 | 1.00 |  |  |  |  |  |  |
| Sex x δ Valence CS^+^ | 0.12 | 0.85 | .397 | 0.80 |  |  |  |  |  |  |
| δ Valence CS^-^ | 0.02 | 0.13 | .894 | 0.99 |  |  |  |  |  |  |
| Sex x δ Valence CS^-^ | 0.01 | 0.04 | .972 | 0.94 |  |  |  |  |  |  |

Results from regression analysis for attentional disengagement from the CS^-^ when presented with the neutral cue. The underscore indicates the appearance of the dot-probe. Significant predictors are indicated by bold font and are given with standardized beta coefficients. Excluded variables are given with unstandardized beta coefficients. For all predictor variables, tolerance (tol.) values from multicollinearity tests are given. For significant interaction terms with sex, results from post-hoc regression analyses are given for women and men separately.

## Table S5

| **δ Attentional disengagement CS^-^/ CS^+^** | | | | | | | | | | |
| --- | --- | --- | --- | --- | --- | --- | --- | --- | --- | --- |
|  | **Full sample** | | | | **Women** | | | **Men** | | |
|  | **β** | **t** | **p** | **tol.** | **β** | **t** | **p** | **β** | **t** | **p** |
| **Sex** | **1.13** | **3.18** | **.002** | **0.11** |  |  |  |  |  |  |
| Trait anxiety | 0.00 | 0.03 | .976 | 0.98 |  |  |  |  |  |  |
| Sex x Trait anxiety | -0.09 | 0.40 | .689 | 0.30 |  |  |  |  |  |  |
| Positive affect | 0.06 | 0.49 | .624 | 1.00 |  |  |  |  |  |  |
| Sex x Positive affect | -0.13 | 0-54 | .594 | 0.28 |  |  |  |  |  |  |
| **Negative affect** | **0.84** | **2.36** | **.022** | **0.10** |  |  |  |  |  |  |
| **Sex x Negative affect** | **-1.54** | **3.19** | **.002** | **0.06** | 0.11 | 0.60 | .555 | -0.60 | 4.12 | < .001 |
| Catastrophizing | 0.08 | 0.71 | .480 | 0.99 |  |  |  |  |  |  |
| Sex x Catastrophizing | 0.07 | 0.55 | .587 | 0.84 |  |  |  |  |  |  |
| Active coping | 0.10 | 0.85 | .398 | 1.00 |  |  |  |  |  |  |
| Sex x Active coping | 0.06 | 0.31 | .755 | 0.35 |  |  |  |  |  |  |
| δ Valence CS^+^ | -0.03 | 0.28 | .781 | 0.99 |  |  |  |  |  |  |
| Sex x δ Valence CS^+^ | -0.01 | 0.05 | .958 | 0.79 |  |  |  |  |  |  |
| δ Valence CS^-^ | -0.09 | 0.75 | .454 | 0.99 |  |  |  |  |  |  |
| Sex x δ Valence CS^-^ | -0.12 | 0.96 | .340 | 0.93 |  |  |  |  |  |  |

Results from regression analysis for attentional disengagement from the CS^-^ when presented with the CS^+^. The underscore indicates the appearance of the dot-probe. Significant predictors are indicated by bold font and are given with standardized beta coefficients. Excluded variables are given with unstandardized beta coefficients. For all predictor variables, tolerance (tol.) values from multicollinearity tests are given. For significant interaction terms with sex, results from post-hoc regression analyses are given for women and men separately.

# Supplementary Figures

## Figure S1


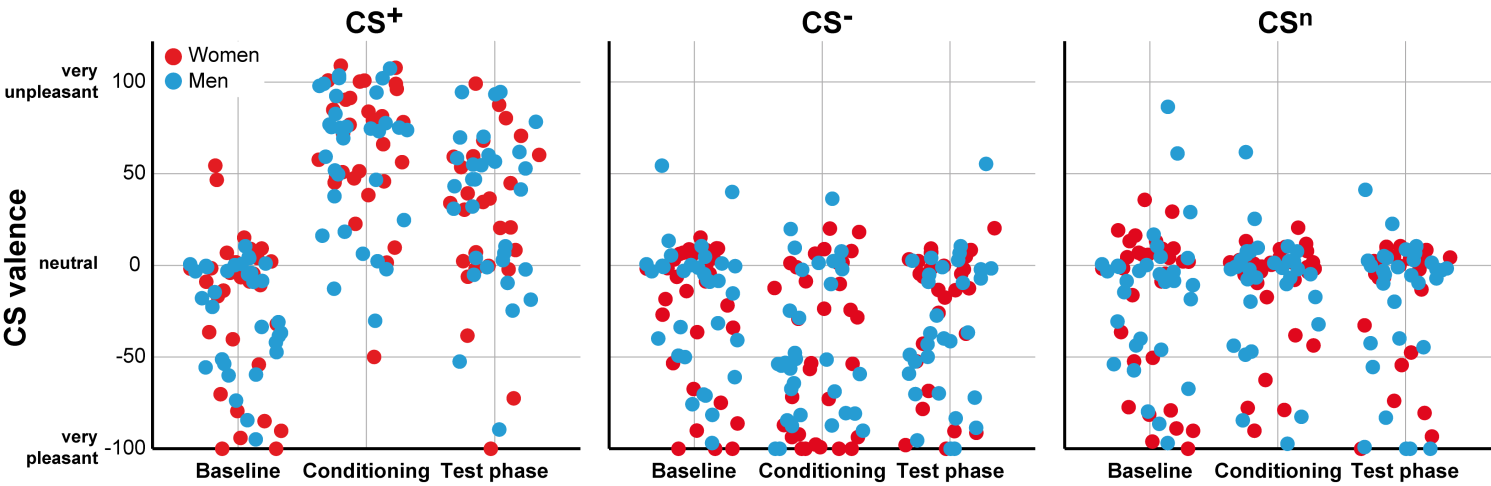


Distribution of single subject data of women (red) and men (blue) for valence ratings of CS^+^, CS^-^ and CS^n^ assessed on visual analogue scales (VAS) before and after conditioning as well as after the test phase.

## Figure S2


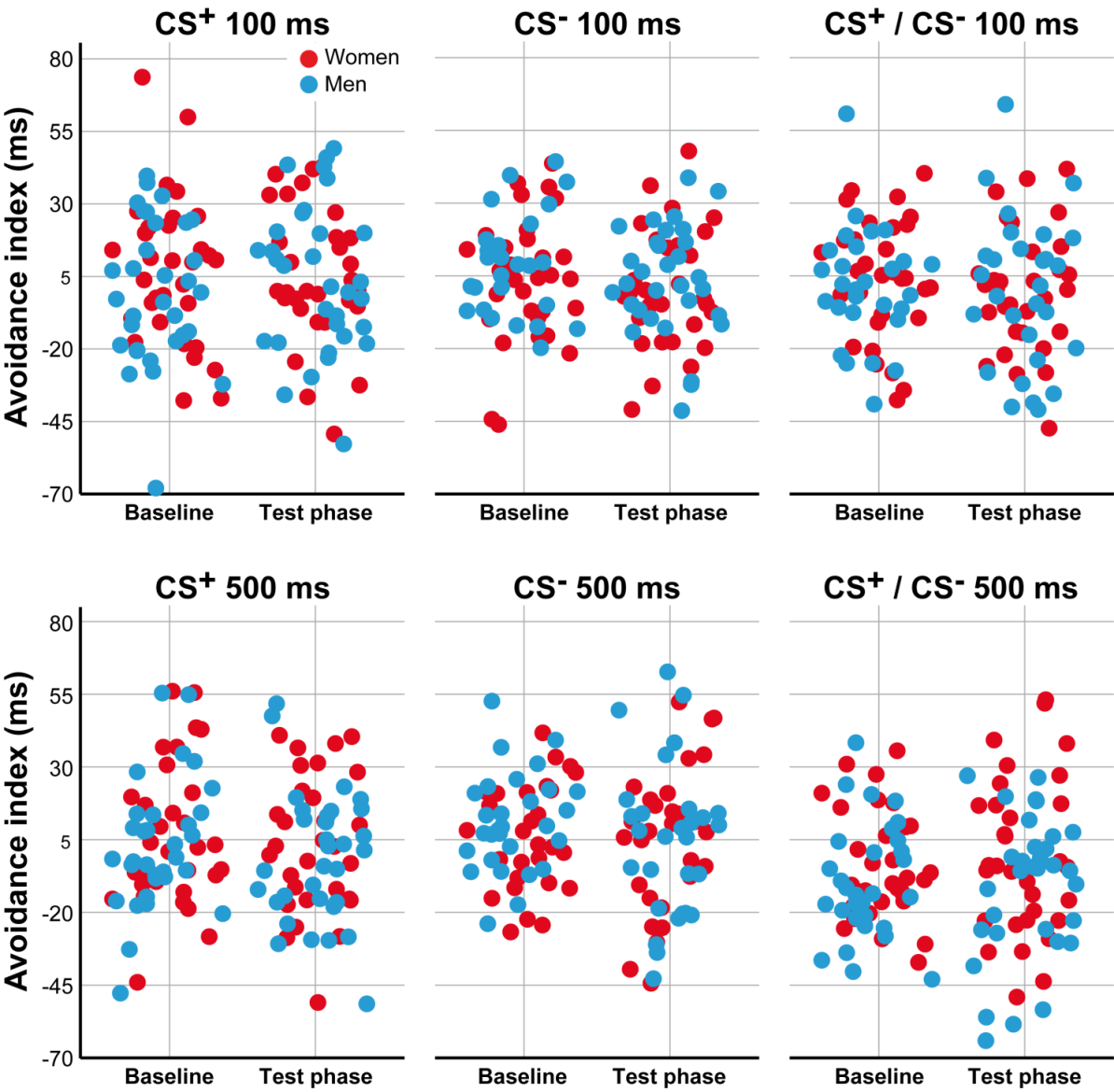


Distribution of single subject data of women (red) and men (blue) for indices of attentional avoidance given in ms for the dot-probe tasks accomplished at baseline and during the test phase. Conditions with 100 ms stimulus duration are given in the upper panel and conditions with 500 ms stimulus duration are given in the lower panel.

## Figure S3


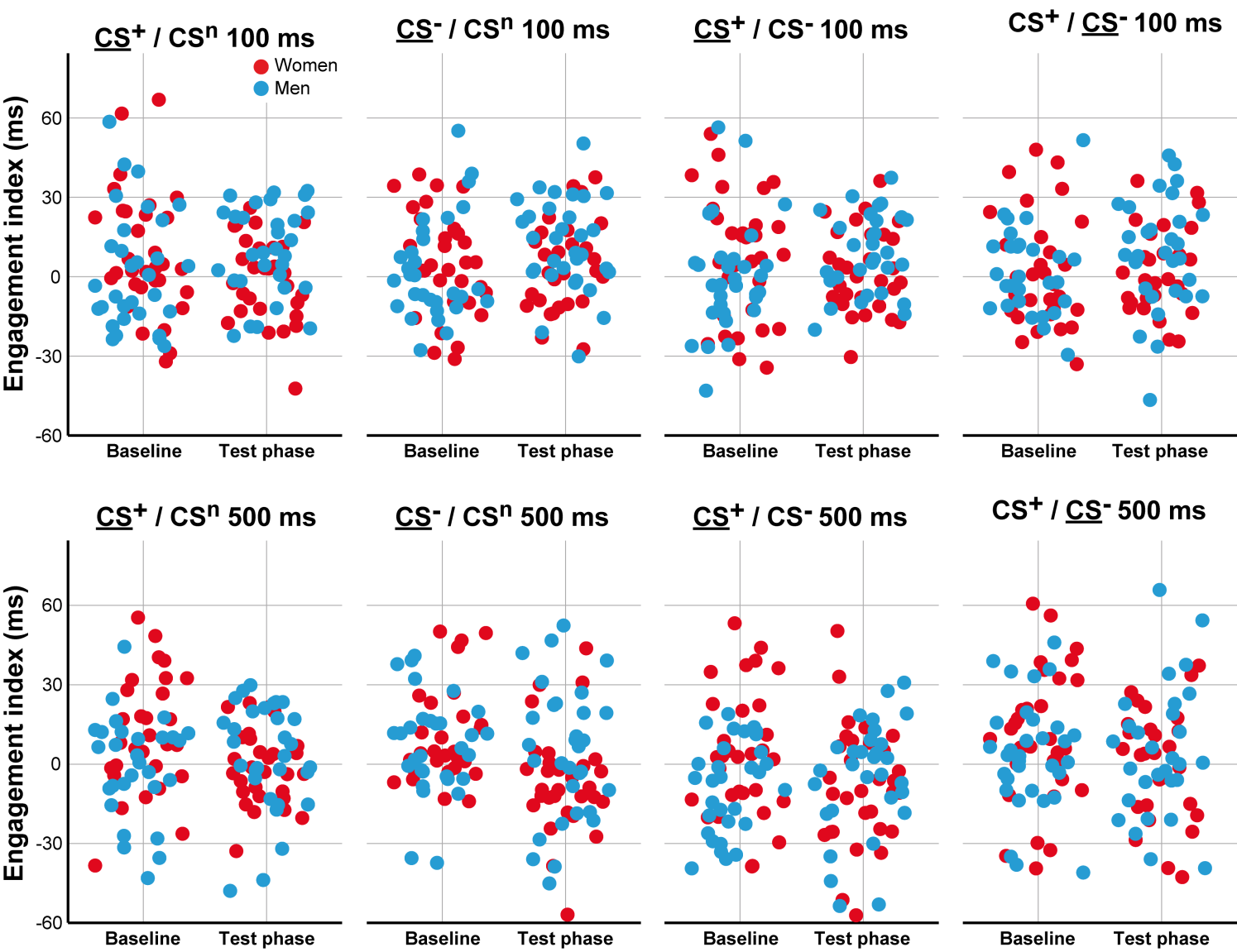


Distribution of single subject data of women (red) and men (blue) for indices of attentional engagement given in ms for the dot-probe tasks accomplished at baseline and during the test phase. Conditions with 100 ms stimulus duration are given in the upper panel and conditions with 500 ms stimulus duration are given in the lower panel. The underscore indicates the location of the dot-probe appearance.

## Figure S4


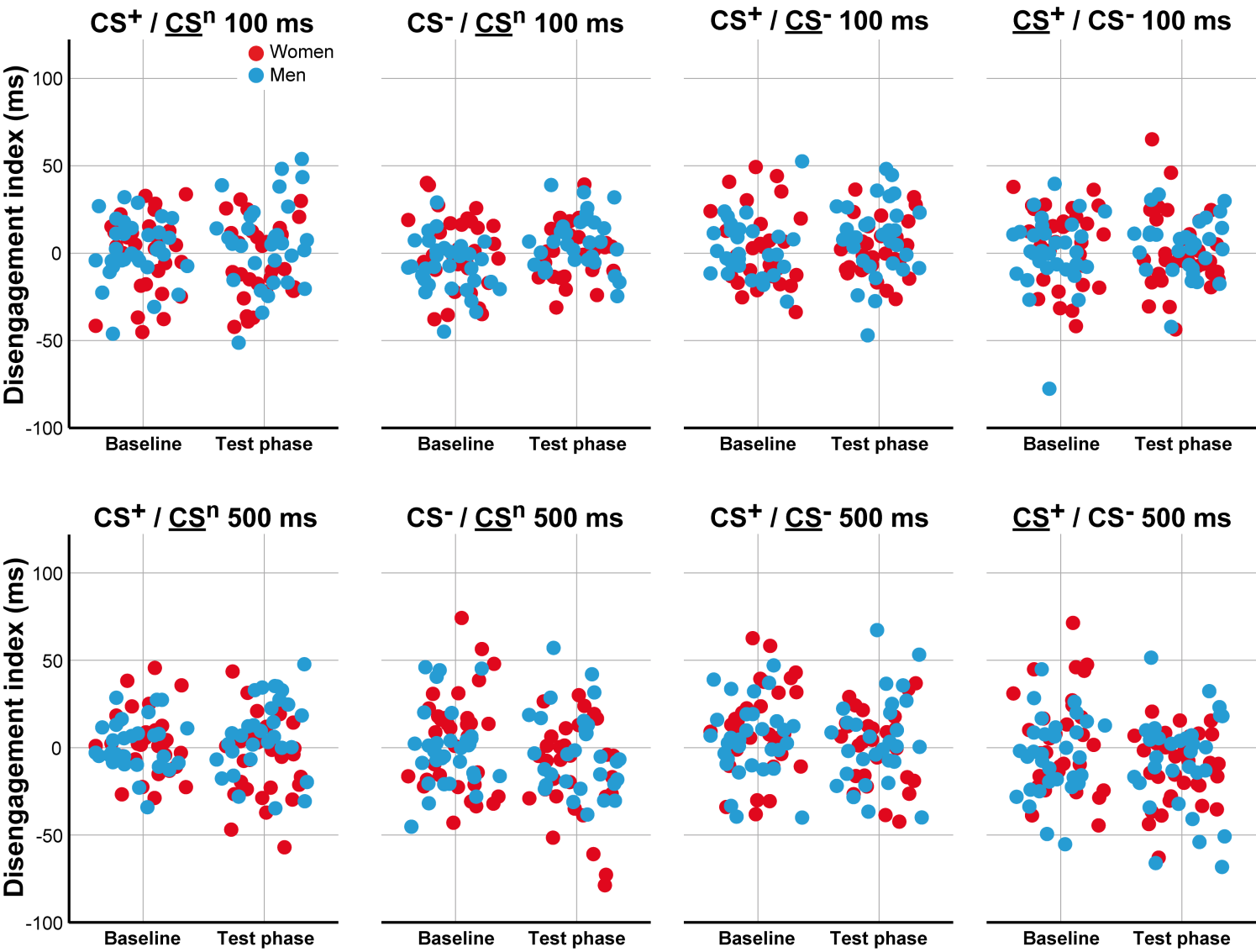


Distribution of single subject data of women (red) and men (blue) for indices of attentional disengagement given in ms for the dot-probe tasks accomplished at baseline and during the test phase. Conditions with 100 ms stimulus duration are given in the upper panel and conditions with 500 ms stimulus duration are given in the lower panel. The underscore indicates the location of the dot-probe appearance.
